# No evidence of a longitudinal association between diurnal cortisol patterns and cognition^☆^

**DOI:** 10.1016/j.neurobiolaging.2014.03.015

**Published:** 2014-10

**Authors:** Archana Singh-Manoux, Aline Dugravot, Alexis Elbaz, Martin Shipley, Mika Kivimaki, Meena Kumari

**Affiliations:** a INSERM, U1018, Centre for Research in Epidemiology and Population Health, Hôpital Paul Brousse Villejuif Cedex, France; b Department of Epidemiology and Public Health, University College London, London, UK; c Centre de Gérontologie, Hôpital Ste Périne, AP-HP, Paris, France; d University Versailles St-Quentin en Yvelines, Paris, France

**Keywords:** Cortisol, Glucocorticoid, Cognitive decline

## Abstract

We examined the effect of salivary cortisol on cognitive performance and decline in 3229 adults (79% men), mean age 61 years. Six saliva samples over the day along with a cognition test battery were administered twice in 5 years. In fully-adjusted cross-sectional analyses from 2002 to 2004, higher waking cortisol was associated with higher reasoning score (β = 0.08, 95% confidence interval: 0.01, 0.15) but this finding was not replicated using data from 2007 to 2009. Over the mean 5 years follow-up there was decline in all cognitive tests but this decline did not vary as a function of cortisol levels; the exception was among APOE e4 carriers where a flatter diurnal slope and higher bedtime cortisol were associated with faster decline in verbal fluency. Changes in cortisol measures between 2002/2004 and 2007/2009 or chronically elevated levels were not associated with cognitive performance in 2007/2009. These results, based on a large sample of community-dwelling adults suggest that variability in hypothalamic-pituitary-adrenal function is not a strong contributor to cognitive aging.

## 1 Introduction

Cortisol is a glucocorticoid hormone, regulated via the hypothalamic-pituitary-adrenal (HPA) axis. Dysregulation of this axis is hypothesized to impair cognitive function, in particular memory and learning processes (Sapolsky et al., 1986, 2000). Much of the evidence for this view comes from animal models where glucocorticoid receptors have been found in the rodent hippocampus (Jacobson and Sapolsky, 1991; McEwen et al., 1986), and elevated glucocorticoid levels shown to be associated with spatial memory impairments and neuronal loss in the hippocampus (Issa et al., 1990; Montaron et al., 2006). Small experimental studies in humans show short term elevations in cortisol improve consolidation of memories (Abercrombie et al., 2003; Buchanan and Lovallo, 2001; Kuhlmann and Wolf, 2006). However, sustained elevations in cortisol may be toxic to brain cells, as in Cushing disease (Starkman et al., 1992) and contribute to risk of dementia. Dementia patients show higher cortisol levels (Elgh et al., 2006; Hartmann et al., 1997) but no disturbance in the diurnal rhythm (Hartmann et al., 1997). Neuroimaging data show plasma cortisol to be correlated with beta amyloid, a hallmark of Alzheimer's disease (Toledo et al., 2012). However, given the long preclinical phase of dementia (Braak and Del, 2011; Jack et al., 2010) it is unclear whether HPA dysregulation is a cause, correlate, or consequence of dementia. Evidence of an association between elevated cortisol levels and accelerated cognitive decline would support an etiological role for cortisol.

There is some evidence, mostly from studies of limited size on elderly persons (Lupien et al., 1994, 1998) that memory is the domain specifically affected by higher cortisol levels. While glucocorticoid receptors are localized to the rodent hippocampus (Jacobson and Sapolsky, 1991; McEwen et al., 1986), they are widely present in primate brains (Sanchez et al., 2000), making it important to consider the association of cortisol with a wide range of cognitive domains. The measurement of cortisol is not straightforward, salivary cortisol over the day is seen to better measure HPA axis function compared with plasma or urinary cortisol (Kirschbaum and Hellhammer, 1989, 1994; Vining et al., 1983). In this article, we examined the association of salivary cortisol, assessed with 6 samples over the day, and cognitive decline over a 5-year period in a large cohort of middle-aged community-dwelling adults. Since APOE e4 is a risk factor for both cognitive decline before the age of 60 years (Caselli et al., 2009) and dementia (Corder et al., 1993) and is hypothesized to modify cortisol's association with cognitive function (Lee et al., 2008), we also conducted analyses stratified by APOE ε4 status. We used tests of memory, reasoning, and verbal fluency in the analysis.

## 2 Methods

### 2.1 Participants

The Whitehall II study is an ongoing study of men and women, originally employed by the British civil service. The target population was all London-based office staff, aged 35–55 years at study inception. A total of 10,308 persons (6895 men and 3413 women), response rate 73%, were recruited to the study in 1985–1988 (Marmot and Brunner, 2005). Since the baseline medical examination, follow-up examinations have taken place approximately every 5 years: 1991/1993 (n = 8815); 1997/1999 (n = 7870); 2002/2004 (n = 6967), and 2007/2009 (n = 6761). Ethical approval for the Whitehall II study was obtained from the University College London Medical School committee on the ethics of human research; all participants provided written informed consent.

### 2.2 Assessment of cortisol, 2002/2004 and 2007/2009

Cortisol data collection was initiated part way through the 2002/2004 wave and repeated in 2007/2009. Instructions were given in a face-to-face interview where participants were requested to provide 6 saliva samples using salivettes over the course of a normal weekday at waking, +30 minutes, +2.5 hours, +8 hours, +12 hours, and bedtime. The intention was to study diurnal rhythm, characterized by high levels upon waking, a further increase–peaking at about 30–40 minutes (the cortisol awakening response, CAR) and subsequent decline over the remainder of the day (Kirschbaum and Hellhammer, 1989). Participants were instructed not to brush their teeth or eat or drink for 15 minutes before sample collection. A booklet was used for participants to record information on the day of sampling including date of collection, wake time, and time each sample was taken. The salivettes and booklet were returned in a prepaid envelope by post. Salivettes were centrifuged at 3000 rpm for 5 minutes, resulting in a clear supernatant of low viscosity. Salivary cortisol levels were measured using a commercial immunoassay with chemiluminescence detection (CLIA; IBLHamburg, Hamburg, Germany). The lower concentration limit of this assay was 0.44 nmol/liter; intra and interassay coefficients of variance were below 8%.

### 2.3 Cognitive function, 2002/2004 and 2007/2009

The cognitive test battery was chosen to provide a comprehensive assessment of cognitive function while being appropriate, with respect to ceiling effects, for this population composed of individuals younger than in most studies on cognitive aging. The tests had high test–retest reliability, range 0.60–0.89, assessed on 556 participants within 3 months of having taken the test in 2002/2004.

Short-term verbal memory was assessed with a free recall test. Participants were presented a list of 20 one or 2 syllable words at 2 seconds intervals and were then asked to recall in writing, within 2 minutes, as many words as possible, in any order.

The Alice Heim 4-I is composed of a series of 65 verbal and mathematical reasoning items of increasing difficulty (Heim, 1970). It tests inductive reasoning, measuring the ability to identify patterns and infer principles and rules. Participants had 10 minutes to do this section.

Verbal fluency (Borkowski et al., 1967), phonemic, and semantic tests were combined for the purposes of the analysis. Participants were asked to recall in writing as many words beginning with “S” (phonemic fluency) and as many animal names (semantic fluency) as they could. One minute was allowed for each test; the observed range on these tests was 0–35.

### 2.4 Covariates

Demographic measures included age, sex, ethnicity (white, non-white), and education, defined as the highest qualification on leaving full-time education and categorized as lower secondary school or less, higher secondary school (usually achieved at the age of 18 years) and university or higher degree. Other covariates in the analyses were seasonality (using 21 March, June, September, and December as cutoffs); depressive symptoms using the 20-item Center for Epidemiologic Studies Depression Scale; (Radloff, 1997) stress on the day of cortisol sampling using questions on whether the participant had experienced a stressful event and, if yes, response to how stressful this was on a 5 point Likert-scale; cardiovascular risk using the Framingham general cardiovascular disease risk score which includes age, sex, systolic blood pressure, treatment for hypertension, high density lipoprotein cholesterol, total cholesterol, smoking, and diabetes (D'Agostino et al., 2008); coronary heart disease and stroke identified using linkage to national hospital records, diabetes mellitus determined by fasting glucose ≥7.0 mmol/L, a 2-hour postload glucose ≥11.1 mmol/L, reported doctor-diagnosed diabetes, or use of diabetes medication; and medication to treat cardiovascular risk factors and depression.

For the sensitivity analysis, we assessed APOE genotype. Two TaqMan assays (Rs429358 and Rs7412, Assay-On-Demand, Applied Biosystems) were used and run on a 7900HT analyzer (Applied Biosystems), and genotypes indicated by the Sequence Detection Software version 2.0 (Applied Biosystems). Participants were categorized as APOE ε4 carriers for those with at least 1 ε4 allele.

### 2.5 Statistical analysis

All analyses were conducted using STATA 12. Participant characteristics were described using percentages, or mean (standard deviation, SD) when appropriate, as a function of sex. Five parameters were used to operationalize diurnal cortisol patterns in the analysis: (1) waking cortisol, the first measure of the day; (2) CAR, cortisol awakening response calculated by subtracting cortisol measured at time 1 (waking) from cortisol measured at time 2 (+30 minutes); (3) diurnal slope to reflect the decline in cortisol levels over the day, calculated by regressing logarithmically transformed cortisol values (excluding cortisol at time 2 so that slope across the day is not unduly biased by CAR) against sample time using a hierarchical linear model (random slope and intercept) where measurement occasion was a level 1 identifier and person a level 2 identifier, lower (more negative) slopes indicate a more rapid decline in cortisol levels, whereas slope values closer to zero reflect flatter diurnal rhythms; (4) bedtime cortisol, the last measure of the day; and (5) mean cortisol over the day, using the area under the curve (Pruessner et al., 2003), this measure was derived using the trapezoid formula and then divided by the time between the first and the last measure of the day for each person to yield mean hourly cortisol.

All 5 measures were categorized into tertiles; we also ran analyses using continuous scores, modeled as an increment of 1 standard deviation after log transforming the values which showed a skewed distribution (waking and bedtime cortisol). Linear regression was used to estimate the cross-sectional and longitudinal association of cortisol measures with cognition, using standardized cognitive test scores (mean = 0; standard deviation = 1). For the analysis of cognitive decline the standardization was undertaken using the 2002/2004 measure, such that the distribution of the baseline was also used to standardize the follow-up data; difference in the standardized scores between the 2 assessments (2007/2009 score–2002/2004 score) was the outcome. These analyses were adjusted for age, sex, ethnicity, education, time of waking, the delay between waking and first cortisol assessment, seasonality, depressive symptoms, stress, Framingham cardiovascular risk score, coronary heart disease, stroke, diabetes, medication for cardiovascular disease (CVD), and antidepressants. Finally, we examined whether changes in cortisol measures between 2002/2004 and 2007/2009 were associated with cognition in 2007/2009. This also included assessing the association of low and high cortisol levels at both time-points with cognitive performance at the second time-point by categorizing participants as being in the first tertile of cortisol distribution at both assessments, being in the third tertile and others.

In sensitivity analyses we reran the cross-sectional analysis using data from the 2007/2009 assessment, undertook analysis of cognitive decline using the growth curve approach (Laird and Ware, 1982), conducted the longitudinal analysis separately in men and women, and finally in analysis stratified by APOE e4 status.

## 3 Results

In 2002/2004, a total of 4498 participants provided cortisol data. After excluding those who reported taking steroid medications (239 corticosteroids and 175 female sex hormones), those with cortisol values outside 3 SD from the mean (n = 91) or incomplete cortisol data (n = 354), we were left with 3736 participants. Those noncomplaint on the cortisol protocol (n = 354) excluded from the analyses, were more likely to be female (26.5% were women compared with 21.3% in the analytic sample, *p* = 0.02) but not age (*p* = 0.30) or education (*p* = 0.16) differences were observed. The main analysis was undertaken on 3229 of these participants with cognitive data in 2002/2004 and 2007/2009. Compared with 1269 (4498 in 2002/2004–3229 in the analysis) participants excluded from the current analyses, the analytic sample consisted of younger (60.8 vs. 61.6 years, *p* < 0.001) participants, more men (78.7% vs. 62.9%, *p* < 0.001), and more educated individuals (30.8% vs. 27.3% had a university degree, *p* = 0.001).

Table 1 presents the sample characteristics of men and women; although there were sex differences in cortisol values we combined men and women in the analysis as the cortisol-cognition associations showed no sex differences (all *p* for interaction >0.05). The cross-sectional association between cortisol and cognitive function at the 2002/2004 assessment (Table 2) show higher waking cortisol to be associated with higher reasoning scores (β = 0.08, 95% confidence interval [CI]: 0.00, 0.15), the same analysis with the 2007/2009 measures (Supplementary Table S1) does not replicate the finding. None of the other associations differed from the null hypothesis of no association (Table 2).

The association between cortisol in 2002/2004 and subsequent cognitive decline are shown in Table 3. Over the mean 5.02 years follow-up there was decline in all cognitive tests, decline in the reference group in the analysis (the lowest cortisol tertile) are shown in Table 3, which also shows that higher cortisol values were not associated with faster cognitive decline. The growth curve approach (Supplementary Table S2) replicated the results obtained using the difference score approach used in Table 3. In analysis stratified by sex (Supplementary Tables S3 and S4), there was some evidence of greater decline in reasoning in women with high bedtime cortisol, no such effects were seen in men.

Supplementary Table S5, presents results of the longitudinal analysis on carries of the APOE e4 allele, these indicate that the flattest slope (β = −0.13, 95% CI: −0.24, −0.02), and the highest bedtime cortisol (β = −0.12, 95% CI: −0.23, −0.01) were associated with faster decline in verbal fluency, no longitudinal associations were observed in noncarrier of the APOE e4 (Supplementary Table S6).

Between 2002/2004 and 2007/2009 waking cortisol declined from a mean of 14.20 to 14.03 nmol/L (*p* < 0.001), CAR from 7.31 to 6.09 nmol/L (*p* < 0.001), mean cortisol from 7.26 to 7.05 nmol/L (*p* = 0.003); the diurnal slope was flatter at the second assessment (−0.131 to −0.116, *p* < 0.001) and bedtime cortisol higher (1.58–1.90 nmol/L, *p* < 0.001). Results presented in Table 4 show no association between change in cortisol measure between 2002/2004 and 2007/2009 and cognitive tests in 2007/2009. Similarly, Table 5 shows that those with elevated levels of cortisol at both assessments had cognitive score similar to those with low cortisol levels.

## 4 Discussion

In this longitudinal analysis of the association between cortisol and cognition in community dwelling participants we found no evidence of a robust association between any feature of the diurnal cortisol pattern or cortisol levels and cognitive decline or change in cortisol levels and cognitive performance. The cross-sectional analyses suggested better cognitive scores in those with higher waking cortisol but these results were not consistent across the 2 data cycles, suggesting that this is a chance finding, perhaps because of the large number of tests undertaken. No associations were observed with memory, either in cross-sectional or longitudinal analyses. Those with elevated cortisol levels at both assessments did not have poorer performance compared with those with low levels, suggesting no evidence of chronic effects.

Most early studies examined the association of cortisol with memory (Lupien et al., 1994, 1998; Seeman et al., 1997) with the focus being on impact of change in cortisol on memory performance. However, these studies were based on small samples, ranging from 11 to 194 older adults; in such samples preclinical dementia could have biased results, as the dementia disease process is known to lead to dysregulation of the HPA axis and hypercortisolemia (Csernansky et al., 2006; Weiner et al., 1997). More recently, an increasingly number of studies has been based on larger samples and assess multiple aspects of cognition. However, these results are far from consistent. Serum cortisol appears not to be associated with cognitive decline (Kalmijn et al., 1998; Schrijvers et al., 2011) or dementia (Schrijvers et al., 2011). One study shows low urinary cortisol, values in the lowest quartile, to be associated with slower cognitive decline (Karlamangla et al., 2005).

Cortisol can be assessed using “average” measures, such as urinary cortisol (Karlamangla et al., 2005; Seeman et al., 1997) or from serum (Kalmijn et al., 1998; MacLullich et al., 2005; Schrijvers et al., 2011). However, salivary cortisol is thought to be a better measure of the biologically active hormone fraction (Kirschbaum and Hellhammer, 1994; Vining et al., 1983), less influenced by physiological and pharmacologic conditions than those based on urine or serum (Kirschbaum and Hellhammer, 1989). Further advantages include the study of the diurnal rhythm as an indicator of HPA axis, healthy functioning being reflected in a strong diurnal pattern (Adam and Kumari, 2009). Small (N < 200) studies on older adults suggest that a flatter slope is associated with poor verbal fluency (Fiocco et al., 2006) and greater decline in memory and executive function (Beluche et al., 2010). Cross-sectional results from the MIDUS study (N = 1500) show healthier cortisol profiles (steeper slope, higher waking, and lower bedtime cortisol) to be associated with better executive function but not memory (Stawski et al., 2011). In another study on 778 middle-aged men, higher overall cortisol output was associated with poorer performance on a range of cognitive tests, including memory; rather surprisingly, in this study lower general cognitive ability at age 20 year was also associated with higher midlife cortisol levels (Franz et al., 2011).

We found only one previous population based study (N = 911) that examined the association of diurnal salivary cortisol with change in cognition and found an unhealthy diurnal profile (lower morning cortisol, higher evening cortisol, and flatter slope) to be associated with faster decline in memory in APOE-ε4 carriers but not in noncarriers and no associations were apparent for executive function and global cognitive score (Gerritsen et al., 2011). In the Rotterdam Study on elderly individuals, APOE genotype did not modify associations of serum cortisol with dementia (Schrijvers et al., 2011). In our study, there was some evidence of a poor diurnal profile being associated with faster decline in verbal fluency (not memory or reasoning) in the APOE ε4 carriers.

The mechanisms underlying heterogeneity in cognitive aging remain poorly understood; HPA axis dysfunction is one of the hypothesized mechanisms with cortisol being a peripheral indicator. However, cortisol-cognition association is not consistent, some studies show no difference in the diurnal cortisol profiles of normal and mildly impaired elderly participants (Wolf et al., 2002), others show higher plasma cortisol to be associated with more rapid increase in symptoms of dementia (Csernansky et al., 2006). It is possible that cortisol-dementia association is subject to the effect of preclinical dementia on HPA axis dysfunction; therefore, the causality issue remains unanswered (Hoschl and Hajek, 2001). Neuroimaging data are also not consistent, one study (N = 99) suggests that higher cortisol is associated with greater beta-amyloid deposition, (Toledo et al., 2012) but another (N = 95) found no association with brain volume (MacLullich et al., 2005). Our results, based on a large sample of community-dwelling persons, suggest that variability in HPA function is not associated with cognitive aging.

Our results must be considered in the context of study strengths and limitations. Data come from a large study, response rate was good, 6 measures over the day were used to study diurnal patterns, the cognitive test battery was suitable for non-elderly populations in that the tests were not affected by threshold effects. However, cortisol was assessed on a single day and therefore subject to imprecision in measurement and contextual effects despite inclusion of a range of covariates in the analysis. A further source of error is adherence to protocol in our study, which was self-reported via a booklet. The cognitive test battery was chosen to examine the determinants of heterogeneity in cognitive aging, not specifically the effects of cortisol; it is possible that other domains of cognition are associated with cortisol. Data collection procedure was cognitively demanding and may have led to analysis on a selected sample. In conclusion, our results show an effect of a flatter slope and higher bedtime cortisol on decline in verbal fluency in those carrying the APOE ε4 allele. However, given the lack of effects on memory and the number of statistical tests undertaken we are moderately confident that this represents a robust finding. There was no evidence that HPA dysfunction is associated with cognitive performance or decline in noncarriers of the APOE ε4 allele.

## Disclosure statement

The authors have no conflicts of interest.

## Figures and Tables

**Table 1:** Sample characteristics in 2002/2004 according to sex

	Men	Women	*p*
N	2541	688	
Age, M (SD)	60.8 (5.8)	61.1 (5.9)	0.25
High education, %	33.8	19.8	<0.001
White, %	95.0	88.8	<0.001
Cortisol			
Waking cortisol (nmol/L), M (SD)^a^	14.29 (1.80)	13.25 (1.81)	0.003
CAR (nmol/L), M (SD)	7.09 (11.19)	8.73 (2.73)	0.05
Diurnal slope (nmol/L), M (SD)	−0.130 (0.025)	−0.133 (0.023)	0.03
Bedtime cortisol (nmol/L), M (SD)^a^	1.57 (2.52)	1.69 (2.43)	0.06
Mean cortisol (nmol/L), M (SD)	7.41 (2.80)	6.71 (2.45)	<0.001
Cognition			
Memory (range 0–20), M (SD)	6.9 (2.3)	7.1 (2.6)	0.11
Reasoning (range 0.65), M (SD)	46.2 (9.6)	40.0 (11.8)	<0.001
Fluency (range 0.35), M (SD)	16.0 (3.3)	15.6 (3.8)	0.02

Key: CAR, cortisol-awakening response; M, mean; SD, standard deviation.

a Geometric mean.

**Table 2:** Cross-sectional association between cortisol and cognitive function, assessed in 2002/2004^a^

Cortisol measures	M (SD) nmol/L	Cognitive function		
		Memory	Reasoning (AH 4-I)	Verbal fluency
		Beta (95% CI)	Beta (95% CI)	Beta (95% CI)
Waking cortisol (tertile)	8.20 (2.65)	REF	REF	REF
	14.88 (1.82)	0.02 (−0.06, 0.10)	0.03 (−0.04, 0.10)	0.00 (−0.08, 0.08)
	25.24 (6.37)	−0.00 (−0.08, 0.08)	0.08 (0.00, 0.15)^b^	−0.01 (−0.09, 0.06)
1 SD increment, log		−0.00 (−0.03, 0.03)	0.02 (−0.01, 0.05)	−0.01 (−0.04, 0.02)
CAR (tertile)	−4.58 (5.91)	REF	REF	REF
	6.55 (2.51)	0.03 (−0.05, 0.11)	0.02 (−0.05, 0.09)	0.06 (−0.01, 0.14)
	19.38 (7.69)	0.05 (−0.03, 0.13)	−0.05 (−0.12, 0.03)	0.06 (−0.02, 0.13)
1 SD increment		0.01 (−0.02, 0.05)	−0.03 (−0.05, 0.00)	0.01 (−0.03, 0.04)
Diurnal slope (tertile)	−0.16 (0.02)	REF	REF	REF
	−0.13 (0.01)	−0.04 (−0.12, 0.04)	0.00 (−0.07, 0.07)	−0.00 (−0.07, 0.08)
	−0.10 (0.01)	−0.06 (−0.15, 0.02)	0.02 (−0.06, 0.09)	−0.05 (−0.13, 0.03)
1 SD increment		−0.02 (−0.06, 0.01)	−0.00 (−0.03, 0.02)	−0.02 (−0.05, 0.01)
Bedtime cortisol (tertile)	0.71 (0.29)	REF	REF	REF
	1.67 (0.32)	−0.06 (−0.14, 0.02)	0.01 (−0.06, 0.08)	−0.00 (−0.07, 0.08)
	5.04 (4.56)	−0.07 (−0.15, 0.01)	−0.01 (−0.08, 0.06)	−0.04 (−0.12, 0.03)
1 SD increment, log		−0.02 (−0.06, 0.01)	−0.01 (−0.04, 0.02)	−0.01 (−0.05, 0.02)
Mean cortisol (tertile)	4.66 (0.96)	REF	REF	REF
	6.85 (0.59)	0.00 (−0.08, 0.08)	0.01 (−0.06, 0.08)	0.03 (−0.04, 0.11)
	10.26 (2.34)	0.01 (−0.07, 0.10)	0.02 (−0.05, 0.09)	0.02 (−0.06, 0.10)
1 SD increment		0.00 (−0.03, 0.04)	0.00 (−0.03, 0.03)	−0.01 (−0.04, 0.02)

Analysis adjusted for age, sex, education, ethnicity, waking time, time between waking and first measure of cortisol, seasonality, depressive symptoms, stress, Framingham cardiovascular risk score, coronary heart disease, stroke, diabetes, medication for cardiovascular disease, and antidepressants. N = 3229. Key: AH 4-I, Alice Heim 4-I; CAR, cortisol-awakening response; CI, confidence interval; M, mean; REF, reference; SD, standard deviation.

a Cognitive data have been converted to z-scores for the analysis.

b *p* < 0.05.

**Table 3:** Cortisol in 2002/2004 as a predictor of cognitive decline between 2002/2004 and 2007/2009^a^

Cortisol measures	M (SD) nmol/L	Cognitive function		
		Memory	Reasoning (AH 4-I)	Verbal fluency
		Beta (95% CI)	Beta (95% CI)	Beta (95% CI)
Waking cortisol (tertile)	8.20 (2.65)	REF^b^_(beta = −0.28)_^c^	REF^b^_(beta = −0.07)_^c^	REF^b^_(beta = −0.16)_^c^
	14.88 (1.82)	0.01 (−0.07, 0.09)	0.04 (0.00, 0.09)^d^	0.01 (−0.05, 0.07)
	25.24 (6.37)	−0.01 (−0.10, 0.07)	−0.00 (−0.04, 0.04)	0.01 (−0.05, 0.06)
1 SD increment, log		−0.01 (−0.04, 0.03)	0.00 (−0.02, 0.02)	0.00 (−0.02, 0.03)
CAR (tertile)	−4.58 (5.90)	REF^b^_(beta = −0.29)_^c^	REF^b^_(beta = −0.06)_^c^	REF^b^_(beta = −0.13)_^c^
	6.55 (2.51)	0.01 (−0.07, 0.10)	0.01 (−0.03, 0.06)	−0.05 (−0.11, 0.01)
	19.38 (7.69)	−0.00 (−0.08, 0.08)	−0.02 (−0.06, 0.02)	−0.03 (−0.09, 0.03)
1 SD increment		0.00 (−0.03, 0.04)	−0.01 (−0.02, 0.01)	−0.01 (−0.03, 0.02)
Diurnal slope (tertile)	−0.16 (0.02)	REF^b^_(beta = −0.30)_^c^	REF^b^_(beta = −0.04)_^c^	REF^b^_(beta = −0.16)_^c^
	−0.13 (0.01)	0.02 (−0.06, 0.10)	−0.02 (−0.06, 0.02)	0.01 (−0.05, 0.07)
	−0.10 (0.01)	0.04 (−0.05, 0.12)	−0.03 (−0.07, 0.01)	−0.01 (−0.06, 0.05)
1 SD increment		0.02 (−0.02, 0.05)	−0.01 (−0.03, 0.01)	−0.00 (−0.02, 0.02)
Bedtime cortisol (tertile)	0.71 (0.29)	REF^b^_(beta = −0.31)_^c^	REF^b^_(beta = −0.05)_^c^	REF^b^_(beta = −0.15)_^c^
	1.67 (0.32)	0.03 (−0.05, 0.11)	−0.01 (−0.06, 0.03)	−0.02 (−0.07, 0.04)
	5.04 (4.56)	0.04 (−0.04, 0.12)	−0.00 (−0.04, 0.04)	−0.00 (−0.06, 0.06)
1 SD increment, log		0.01 (−0.02, 0.05)	−0.01 (−0.02, 0.01)	−0.01 (−0.03, 0.02)
Mean cortisol (tertile)	4.66 (0.96)	REF^b^_(beta = −0.27)_^c^	REF^b^_(beta = −0.04)_^c^	REF^b^_(beta = −0.14)_^c^
	6.85 (0.59)	−0.06 (−0.14, 0.02)	−0.03 (−0.07, 0.02)	−0.03 (−0.08, 0.03)
	10.26 (2.34)	0.02 (−0.06, 0.11)	−0.04 (−0.08, 0.00)	−0.03 (−0.09, 0.03)
1 SD increment		0.00 (−0.03, 0.04)	−0.02 (−0.03, 0.00)	−0.01 (−0.03, 0.01)

N = 3229. Key: AH 4-I, Alice Heim 4-I; CAR, cortisol-awakening response; CI, confidence interval; M, mean; REF, reference; SD, standard deviation.

a Cognitive data have been converted to z-scores for the analysis.

b Reference category; subscript beta shows the adjusted standardized cognitive decline in the reference category analysis adjusted for age, sex, education, ethnicity, waking time, time between waking and first measure of cortisol, seasonality, depressive symptoms, stress, Framingham cardiovascular risk score, coronary heart disease, stroke, diabetes, CVD medication, and antidepressants.

c *p* < 0.01.

d *p* < 0.05.

**Table 4:** Change in cortisol between 2002/2004 and 2007/2009 and cognitive function in 2007/2009^a^

Change in cortisol measure	M (SD) nmol/L	Cognitive function		
		Memory	Reasoning (AH 4-I)	Verbal fluency
		Beta (95% CI)	Beta (95% CI)	Beta (95% CI)
Waking cortisol (tertile)	−11.51 (6.23)	REF	REF	REF
	−0.97 (1.99)	0.05 (−0.04, 0.14)	0.05 (−0.03, 0.13)	0.04 (−0.05, 0.12)
	9.15 (6.44)	0.02 (−0.07, 0.11)	−0.00 (−0.08, 0.07)	0.03 (−0.06, 0.11)
1 SD increment, log		0.01 (−0.02, 0.04)	−0.00 (−0.03, 0.02)	0.02 (−0.00, 0.05)
CAR (tertile)	−16.62 (9.21)	REF	REF	REF
	−1.44 (2.95)	0.04 (−0.05, 0.13)	0.05 (−0.03, 0.13)	−0.03 (−0.11, 0.06)
	14.15 (9.28)	−0.03 (−0.12, 0.06)	0.06 (−0.02, 0.13)	−0.02 (−0.11, 0.06)
1 SD increment		−0.01 (−0.04, 0.02)	0.02 (−0.00, 0.05)	−0.00 (−0.03, 0.02)
Diurnal slope (tertile)	−0.01 (0.02)	REF	REF	REF
	0.01 (0.01)	0.03 (−0.06, 0.12)	−0.00 (−0.08, 0.07)	0.01 (−0.08, 0.09)
	0.04 (0.02)	−0.00 (−0.09, 0.09)	0.01 (−0.07, 0.09)	0.02 (−0.07, 0.11)
1 SD increment		0.00 (−0.03, 0.04)	0.01 (−0.02, 0.04)	0.01 (−0.02, 0.04)
Bedtime cortisol (tertile)	−2.72 (4.20)	REF	REF	REF
	0.32 (0.35)	0.01 (−0.08, 0.10)	0.03 (−0.04, 0.11)	0.01 (−0.08, 0.09)
	3.24 (3.66)	−0.00 (−0.09, 0.09)	0.04 (−0.04, 0.12)	0.03 (−0.06, 0.12)
1 SD increment, log		−0.00 (−0.03, 0.03)	0.02 (−0.01, 0.04)	0.01 (−0.02, 0.04)
Mean cortisol (tertile)	−3.75 (2.23)	REF	REF	REF
	−0.28 (0.71)	−0.04 (−0.13, 0.05)	0.00 (−0.07, 0.08)	0.08 (−0.01, 0.17)
	3.38 (2.35)	0.02 (−0.07, 0.10)	0.01 (−0.06, 0.09)	0.01 (−0.08, 0.09)
1 SD increment		−0.00 (−0.03, 0.03)	0.00 (−0.02, 0.03)	0.00 (−0.03, 0.03)

Analysis adjusted for age, sex, education, ethnicity, waking time, time between waking and first measure of cortisol, seasonality, depressive symptoms, stress, Framingham cardiovascular risk score, coronary heart disease, stroke, diabetes, CVD medication, and antidepressants. N = 2553. Key: AH 4-I, Alice Heim 4-I; CAR, cortisol awakening response; CI, confidence interval; M, mean; REF, reference; SD, standard deviation.

a Cognitive data have been converted to z-scores for the analysis.

**Table 5:** Chronicity of cortisol levels in 2002/2004 and 2007/2009 and cognitive function in 2007/2009^a^

Cortisol measures	N	Cognitive decline		
		Memory	Reasoning (AH 4-I)	Verbal fluency
		Beta (95% CI)	Beta (95% CI)	Beta (95% CI)
Waking cortisol				
First tertile in 2002/2004 and 2007/2009	331	REF	REF	REF
Others	1834	0.01 (−0.10, 0.12)	−0.01 (−0.11, 0.09)	0.03 (−0.08, 0.13)
Third tertile in 2002/2004 and 2007/2009	388	0.01 (−0.13, 0.15)	0.02 (−0.11, 0.14)	−0.00 (−0.14, 0.13)
CAR				
First tertile in 2002/2004 and 2007/2009	313	REF	REF	REF
Others	1883	0.05 (−0.07, 0.16)	0.02 (−0.08, 0.11)	0.13 (0.02, 0.23)^b^
Third tertile in 2002/2004 and 2007/2009	357	0.12 (−0.02, 0.26)	−0.01 (−0.13, 0.11)	0.09 (−0.04, 0.23)
Diurnal slope				
First tertile in 2002/2004 and 2007/2009	379	REF	REF	REF
Others	1812	−0.01 (−0.12, 0.09)	−0.04 (−0.13, 0.05)	0.04 (−0.06, 0.14)
Third tertile in 2002/2004 and 2007/2009	362	−0.05 (−0.19, 0.08)	−0.07 (−0.19, 0.05)	−0.05 (−0.18, 0.08)
Bedtime cortisol				
First tertile in 2002/2004 and 2007/2009	369	REF	REF	REF
Others	1804	−0.01 (−0.12, 0.10)	−0.00 (−0.09, 0.09)	−0.02 (−0.12, 0.08)
Third tertile in 2002/2004 and 2007/2009	380	−0.10 (−0.23, 0.04)	−0.04 (−0.16, 0.08)	−0.07 (−0.20, 0.06)
Mean cortisol				
First tertile in 2002/2004 and 2007/2009	359	REF	REF	REF
Others	1846	0.05 (−0.05, 0.16)	0.05 (−0.04, 0.14)	0.01 (−0.09, 0.11)
Third tertile in 2002/2004 and 2007/2009	348	0.02 (−0.12, 0.16)	0.01 (−0.12, 0.13)	−0.04 (−0.18, 0.09)

Analysis adjusted for age, sex, education, ethnicity, waking time, time between waking and first measure of cortisol, seasonality, depressive symptoms, stress, Framingham cardiovascular risk score, coronary heart disease, stroke, diabetes, CVD medication, and antidepressants. N = 2553. Key: AH 4-I, Alice Heim 4-I; CAR, cortisol-awakening response; CI, confidence interval; REF, reference.

a Cognitive data have been converted to z-scores for the analysis.

b *p* < 0.05.
