# Supplementary material for: No evidence of a longitudinal association between diurnal cortisol patterns and cognition
Source: Neurobiol Aging. 2014 Oct;35(10):2239–45. doi: 10.1016/j.neurobiolaging.2014.03.015 (PMC4099515; doi:10.1016/j.neurobiolaging.2014.03.015)
Supplement: Supplementary Tables S1–S6 [file mmc1.docx]

**SENSITIVITY ANALYSIS**

Table S1. Cross-sectional association between cortisol and cognitive function, assessed in 2007/09.^a^

| **Cortisol measure** | **Cognitive function** | | |
| --- | --- | --- | --- |
|  | **Memory** | **Reasoning (AH 4-I)** | **Verbal Fluency** |
|  | Beta (95% CI) | Beta (95% CI) | Beta (95% CI) |
| **Waking Cortisol**  (tertile) | ref | ref | ref |
|  | -0.00 (-0.07, 0.07) | -0.02 (-0.08, 0.04) | 0.06 (-0.01, 0.12) |
|  | -0.02 (-0.09, 0.05) | 0.01 (-0.05, 0.07) | 0.05 (-0.02, 0.11) |
| *1 SD increment, log* | *0.00 (-0.03, 0.03)* | *0.02 (-0.01, 0.04)* | *0.02 (-0.00, 0.05)* |
| **CAR**  (tertile) | ref | ref | Ref |
|  | -0.00 (-0.07, 0.07) | 0.02 (-0.04, 0.08) | 0.05 (-0.02, 0.11) |
|  | 0.05 (-0.02, 0.12) | 0.04 (-0.02, 0.10) | 0.02 (-0.05, 0.08) |
| *1 SD increment* | *0.02 (-0.01, 0.05)* | *0.02 (-0.01, 0.04)* | *0.01 (-0.02, 0.03)* |
| **Slope**  (tertile) | ref | ref | ref |
|  | 0.02 (-0.05, 0.09) | 0.04 (-0.02, 0.10) | 0.08 (0.01, 0.14)* |
|  | -0.01 (-0.09, 0.06) | -0.01 (-0.07, 0.05) | 0.03 (-0.04, 0.10) |
| *1 SD increment* | *0.00 (-0.03, 0.03)* | *-0.00 (-0.03, 0.02)* | *0.01 (-0.02, 0.03)* |
| **Bedtime Cortisol**  (tertile) | ref | ref | ref |
|  | 0.04 (-0.03, 0.11) | 0.02 (-0.04, 0.08) | 0.06 (-0.00, 0.13) |
|  | -0.04 (-0.12, 0.03) | 0.00 (-0.06, 0.06) | 0.02 (-0.05, 0.08) |
| *1 SD increment, log* | *-0.01 (-0.04, 0.02)* | *-0.00 (-0.03, 0.02)* | *-0.01 (-0.03, 0.02)* |
| **Mean Cortisol**  (tertile) | ref | ref | ref |
|  | 0.03 (-0.04, 0.10) | 0.04 (-0.02, 0.10) | 0.08 (0.01, 0.14)* |
|  | -0.00 (-0.07, 0.07) | 0.03 (-0.03, 0.09) | 0.03 (-0.03, 0.10) |
| *1 SD increment* | *-0.00 (-0.03, 0.03)* | *0.01 (-0.01, 0.04)* | *0.00 (-0.03, 0.03)* |

^a^ Cognitive data have been converted to z-scores for the analysis.

Analysis adjusted for age, sex, education, ethnicity, waking time, time between waking and first measure of cortisol, seasonality, depressive symptoms, stress, Framingham cardiovascular risk score, coronary heart disease, stroke, diabetes, CVD medication, and antidepressants.

N=4233

*p<0.05

Table S2. Cortisol in 2002/04 as a predictor of cognitive decline between 2002/04 and 2007/09 using growth curve modelling. ^a^

| **Cortisol measures** | M (SD) nmol/L | **Cognitive function** | | |
| --- | --- | --- | --- | --- |
|  |  | **Memory** | **Reasoning (AH 4-I)** | **Verbal Fluency** |
|  |  | Beta (95% CI) | Beta (95% CI) | Beta (95% CI) |
| **Waking Cortisol**  (tertile) | 8.20 (2.65) | ref | ref | ref |
|  | 14.88 (1.82) | -0.00 (-0.16, 0.16) | 0.09 (0.00, 0.17)* | 0.01 (-0.10, 0.13) |
|  | 25.24 (6.37) | -0.05 (-0.21, 0.11) | 0.01 (-0.08, 0.09) | -0.00 (-0.11, 0.11) |
| *1 SD increment, log* | | *-0.02 (-0.08, 0.05)* | *0.01 (-0.03, 0.04)* | *0.00 (-0.05, 0.05)* |
| **CAR**  (tertile) | -4.58 (5.90) | ref | ref | ref |
|  | 6.55 (2.51) | 0.02 (-0.14, 0.18) | 0.02 (-0.06, 0.11) | -0.09 (-0.20, 0.03) |
|  | 19.38 (7.69) | -0.02 (-0.18, 0.15) | -0.05 (-0.13, 0.04) | -0.04 (-0.15, 0.07) |
| *1 SD increment* | | *0.00 (-0.03, 0.04)* | *-0.02 (-0.05, 0.02)* | *-0.01 (-0.05, 0.04)* |
| **Diurnal Slope**  (tertile) | -0.16 (0.02) | ref | ref | ref |
|  | -0.13 (0.01) | 0.03 (-0.13, 0.19) | -0.04 (-0.13, 0.04) | 0.02 (-0.09, 0.13) |
|  | -0.10 (0.01) | 0.05 (-0.11, 0.21) | -0.07 (-0.15, 0.01) | -0.04 (-0.15, 0.07) |
| *1 SD increment* | | *0.02 (-0.05, 0.08)* | *-0.03 (-0.06, 0.01)* | *-0.01 (-0.06, 0.03)* |
| **Bedtime Cortisol**  (tertile) | 0.71 (0.29) | ref | ref | ref |
|  | 1.67 (0.32) | 0.05 (-0.11, 0.21) | -0.04 (-0.12, 0.05) | -0.04 (-0.15, 0.08) |
|  | 5.04 (4.56) | 0.08 (-0.09, 0.24) | -0.02 (-0.10, 0.07) | -0.01 (-0.12, 0.10) |
| *1 SD increment, log* | | *0.02 (-0.05, 0.09)* | *-0.02 (-0.05, 0.02)* | *-0.02 (-0.06, 0.03)* |
| **Mean Cortisol**  (tertile) | 4.66 (0.96) | ref | ref | ref |
|  | 6.85 (0.59) | -0.14 (-0.30, 0.03) | -0.05 (-0.14, 0.03) | -0.06 (-0.17, 0.06) |
|  | 10.26 (2.34) | 0.01 (-0.15, 0.17) | -0.08 (-0.16, 0.00) | -0.07 (-0.18, 0.04) |
| *1 SD increment* | | *-0.01 (-0.08, 0.06)* | *-0.03 (-0.07, 0.00)* | *-0.02 (-0.07, 0.02)* |

^a^ Cognitive data have been converted to z-scores for the analysis.

Analysis adjusted for age, sex, education, ethnicity, waking time, time between waking and first measure of cortisol, seasonality, depressive symptoms, stress, Framingham cardiovascular risk score, coronary heart disease, stroke, diabetes, CVD medication, and antidepressants.

M Mean, SD Standard Deviation; N=3229

*p<0.05

Table S3. Cortisol in 2002/04 as a predictor of cognitive decline between 2002/04 and 2007/09 in MEN, N=2541.^a^

|  |  | **Cognitive decline** | | |
| --- | --- | --- | --- | --- |
|  |  | **Memory** | **Reasoning (AH 4-I)** | **Verbal Fluency** |
| **Cortisol measures** |  | Beta (95% CI) | Beta (95% CI) | Beta (95% CI) |
| **Waking Cortisol**  (tertile) |  | ref | ref | ref |
|  |  | 0.01 (-0.09, 0.10) | 0.03 (-0.02, 0.08) | -0.00 (-0.07, 0.06) |
|  |  | -0.01 (-0.11, 0.08) | 0.01 (-0.04, 0.06) | 0.02 (-0.05, 0.08) |
| *1 SD increment, log* |  | *-0.01 (-0.05, 0.03)* | *0.00 (-0.02, 0.02)* | *0.00 (-0.02, 0.03)* |
| **CAR**  (tertile) |  | ref | ref | ref |
|  |  | 0.03 (-0.06, 0.12) | 0.01 (-0.03, 0.06) | -0.06 (-0.12, 0.01) |
|  |  | 0.01 (-0.08, 0.10) | -0.02 (-0.07, 0.03) | -0.04 (-0.10, 0.03) |
| *1 SD increment* |  | *0.00 (-0.04, 0.04)* | *-0.00 (-0.02, 0.01)* | *-0.01 (-0.03, 0.02)* |
| **Diurnal Slope**  (tertile) |  | ref | ref | ref |
|  |  | 0.02 (-0.07, 0.11) | -0.02 (-0.07, 0.03) | 0.01 (-0.07, 0.05) |
|  |  | 0.06 (-0.03, 0.15) | -0.01 (-0.06, 0.03) | -0.03 (-0.09, 0.04) |
| *1 SD increment* |  | *0.03 (-0.01, 0.07)* | *-0.00 (-0.02, 0.02)* | *-0.01 (-0.03, 0.02)* |
| **Bedtime Cortisol**  (tertile) |  | ref | ref | ref |
|  |  | 0.07 (-0.02, 0.16) | -0.01 (-0.06, 0.03) | -0.04 (-0.11, 0.02) |
|  |  | 0.06 (-0.03, 0.15) | 0.02 (-0.03, 0.07) | -0.01 (-0.08, 0.05) |
| *1 SD increment, log* |  | *0.02 (-0.01, 0.06)* | *-0.01 (-0.01, 0.03)* | *-0.02 (-0.04, 0.01)* |
| **Mean Cortisol** (tertile) |  | ref | ref | ref |
|  |  | 0.00 (-0.09, 0.10) | -0.04 (-0.08, 0.01) | -0.05 (-0.12, 0.01) |
|  |  | 0.06 (-0.03, 0.16) | -0.05 (-0.10,0.00) | -0.02 (-0.09, 0.04) |
| *1 SD increment* |  | *0.01 (-0.03, 0.05)* | *-0.02 (-0.04, 0.00)* | *-0.01 (-0.04, 0.02)* |

^a^ Cognitive data have been converted to z-scores for the analysis.

Analysis adjusted for age, sex, education, ethnicity, waking time, time between waking and first measure of cortisol, seasonality, depressive symptoms, stress, Framingham cardiovascular risk score, coronary heart disease, stroke, diabetes, CVD medication, and antidepressants.

*p<0.05

Table S4. Cortisol in 2002/04 as a predictor of cognitive decline between 2002/04 and 2007/09 in WOMEN, N=688.^a^

|  |  | **Cognitive decline** | | |
| --- | --- | --- | --- | --- |
|  |  | **Memory** | **Reasoning (AH 4-I)** | **Verbal Fluency** |
| **Cortisol measures** |  | Beta (95% CI) | Beta (95% CI) | Beta (95% CI) |
| **Waking Cortisol**  (tertile) |  | ref | ref | ref |
|  |  | 0.00 (-0.18, 0.19) | 0.10 (0.01, 0.19)* | 0.05 (-0.07, 0.17) |
|  |  | -0.01 (-0.20, 0.19) | -0.04 (-0.13, 0.05) | -0.03 (-0.15, 0.10) |
| *1 SD increment, log* |  | *0.02 (-0.06, 0.10)* | *-0.00 (-0.05, 0.03)* | *-0.00 (-0.05, 0.05)* |
| **CAR**  (tertile) |  | ref | ref | ref |
|  |  | -0.02 (-0.23, 0.18) | 0.01 (-0.08, 0.11) | -0.02 (-0.15, 0.11) |
|  |  | -0.03 (-0.23, 0.16) | 0.00 (-0.09, 0.10) | -0.00 (-0.13, 0.12) |
| *1 SD increment* |  | *0.01 (-0.06, 0.09)* | *-0.01 (-0.04, 0.03)* | *0.03 (-0.03, 0.08)* |
| **Diurnal Slope**  (tertile) |  | ref | ref | ref |
|  |  | -0.04 (-0.23, 0.14) | 0.00 (-0.08, 0.09) | 0.08 (-0.04, 0.19) |
|  |  | -0.07 (-0.27, 0.13) | -0.10 (-0.19, 0.00) | 0.09 (-0.04, 0.22) |
| *1 SD increment* |  | *-0.04 (-0.13, 0.04)* | *-0.01 (-0.03, 0.01)* | *-0.00 (-0.02, 0.02)* |
| **Bedtime Cortisol**  (tertile) |  | ref | ref | ref |
|  |  | -0.15 (-0.34, 0.05) | -0.02 (-0.11, 0.07) | 0.08 (-0.05, 0.20) |
|  |  | -0.04 (-0.23, 0.16) | -0.09 (-0.18, 0.00) | 0.05 (-0.07, 0.17) |
| *1 SD increment, log* |  | *-0.04 (-0.12, 0.04)* | *-0.06 (-0.10, -0.03)** | *0.02 (-0.03, 0.07)* |
| **Mean Cortisol** (tertile) |  | ref | ref | ref |
|  |  | -0.28 (-0.47, -0.10)* | 0.02 (-0.07, 0.10) | 0.06 (-0.06, 0.18) |
|  |  | -0.09 (-0.29, 0.11) | -0.02 (-0.12,0.07) | -0.07 (-0.20, 0.06) |
| *1 SD increment* |  | *-0.03 (-0.12, 0.06)* | *-0.01 (-0.05, 0.03)* | *-0.01 (-0.07, 0.05)* |

^a^ Cognitive data have been converted to z-scores for the analysis.

Analysis adjusted for age, sex, education, ethnicity, waking time, time between waking and first measure of cortisol, seasonality, depressive symptoms, stress, Framingham cardiovascular risk score, coronary heart disease, stroke, diabetes, CVD medication, and antidepressants.

*p<0.05

Table S5. Cortisol in 2002/04 as a predictor of cognitive decline between 2002/04 and 2007/09 in carriers of the APOE ε4 allele, N=836.^a^

|  |  | **Cognitive decline** | | |
| --- | --- | --- | --- | --- |
|  |  | **Memory** | **Reasoning (AH 4-I)** | **Verbal Fluency** |
| **Cortisol measures** |  | Beta (95% CI) | Beta (95% CI) | Beta (95% CI) |
| **Waking Cortisol**  (tertile) |  | ref | ref | ref |
|  |  | 0.08 (-0.09, 0.26) | 0.04 (-0.05, 0.13) | -0.07 (-0.18, 0.04) |
|  |  | 0.02 (-0.16, 0.20) | -0.03 (-0.12, 0.06) | -0.09 (-0.21, 0.02) |
| *1 SD increment, log* |  | *-0.01 (-0.08, 0.06)* | *-0.01 (-0.05, 0.02)* | *-0.01 (-0.06, 0.03)* |
| **CAR**  (tertile) |  | ref | ref | ref |
|  |  | -0.03 (-0.20, 0.14) | -0.03 (-0.11, 0.06) | -0.03 (-0.14, 0.08) |
|  |  | 0.07 (-0.10, 0.24) | 0.03 (-0.05, 0.12) | -0.04 (-0.15, 0.06) |
| *1 SD increment* |  | *0.02 (-0.04, 0.09)* | *0.00 (-0.03, 0.04)* | *-0.02 (-0.06, 0.03)* |
| **Diurnal Slope**  (tertile) |  | ref | ref | ref |
|  |  | 0.02 (-0.14, 0.19) | -0.02 (-0.10, 0.07) | 0.00 (-0.11, 0.11) |
|  |  | 0.01 (-0.16, 0.18) | 0.01 (-0.10, 0.08) | -0.13 (-0.24,-0.02)* |
| *1 SD increment* |  | *0.01 (-0.06, 0.08)* | *-0.00 (-0.04, 0.04)* | *-0.03 (-0.07, 0.02)* |
| **Bedtime Cortisol**  (tertile) |  | ref | ref | ref |
|  |  | -0.02 (-0.18, 0.15) | 0.04 (-0.05, 0.12) | -0.03 (-0.13, 0.08) |
|  |  | -0.03 (-0.20, 0.14) | 0.03 (-0.06, 0.11) | -0.12 (-0.23,-0.01)* |
| *1 SD increment, log* |  | *0.00 (-0.07, 0.07)* | *0.01 (-0.02, 0.04)* | *-0.03 (-0.07, 0.02)* |
| **Mean Cortisol** (tertile) |  | ref | ref | ref |
|  |  | -0.03 (-0.20, 0.14) | -0.08 (-0.16, 0.01) | -0.06 (-0.17, 0.04) |
|  |  | 0.07 (-0.10, 0.24) | -0.06 (-0.15, 0.02) | -0.07 (-0.18, 0.04) |
| *1 SD increment* |  | *0.01 (-0.06, 0.08)* | *-0.02 (-0.05, 0.02)* | *-0.02 (-0.07, 0.02)* |

^a^ Cognitive data have been converted to z-scores for the analysis.

Analysis adjusted for age, sex, education, ethnicity, waking time, time between waking and first measure of cortisol, seasonality, depressive symptoms, stress, Framingham cardiovascular risk score, coronary heart disease, stroke, diabetes, CVD medication, and antidepressants.

*p<0.05

Table S6. Cortisol in 2002/04 as a predictor of cognitive decline between 2002/04 and 2007/09 in **non-carriers** of the APOE ε4 allele, N=2241.^a^

|  |  | **Cognitive decline** | | |
| --- | --- | --- | --- | --- |
|  |  | **Memory** | **Reasoning (AH 4-I)** | **Verbal Fluency** |
| **Cortisol measures** |  | Beta (95% CI) | Beta (95% CI) | Beta (95% CI) |
| **Waking Cortisol**  (tertile) |  | ref | ref | ref |
|  |  | -0.02 (-0.12, 0.08) | 0.03 (-0.01, 0.09) | 0.04 (-0.03, 0.11) |
|  |  | -0.02 (-0.12, 0.08) | 0.01 (-0.04, 0.06) | 0.05 (-0.02, 0.12) |
| *1 SD increment, log* |  | *-0.00 (-0.04, 0.04)* | *0.01 (-0.02, 0.03)* | *0.01 (-0.01, 0.04)* |
| **CAR**  (tertile) |  | ref | ref | ref |
|  |  | 0.02 (-0.08, 0.12) | 0.04 (-0.01, 0.09) | -0.06 (-0.13, 0.01) |
|  |  | -0.03 (-0.13, 0.07) | -0.03 (-0.08, 0.02) | -0.04 (-0.11, 0.03) |
| *1 SD increment* |  | *-0.01 (-0.05, 0.03)* | *-0.00 (-0.03, 0.02)* | *-0.00 (-0.03, 0.02)* |
| **Diurnal Slope**  (tertile) |  | ref | ref | ref |
|  |  | 0.05 (-0.05, 0.14) | -0.01 (-0.06, 0.04) | 0.03 (-0.04, 0.10) |
|  |  | 0.08 (-0.02, 0.18) | -0.04 (-0.09, 0.01) | 0.04 (-0.03,0.11) |
| *1 SD increment* |  | *0.03 (-0.01, 0.07)* | *-0.02 (-0.04, 0.01)* | *0.01 (-0.02, 0.04)* |
| **Bedtime Cortisol**  (tertile) |  | ref | ref | ref |
|  |  | 0.06 (-0.03, 0.16) | 0.03 (-0.08, 0.02) | -0.00 (-0.07, 0.07) |
|  |  | 0.08 (-0.02, 0.17) | 0.02 (-0.07, 0.03) | 0.05 (-0.02, 0.11) |
| *1 SD increment, log* |  | *0.02 (-0.02, 0.06)* | *-0.01 (-0.03, 0.01)* | *0.00 (-0.03, 0.03)* |
| **Mean Cortisol** (tertile) |  | ref | ref | ref |
|  |  | -0.07 (-0.17, 0.03) | -0.01 (-0.06, 0.04) | -0.01 (-0.08, 0.06) |
|  |  | 0.03 (-0.07, 0.13) | -0.03 (-0.08, 0.02) | -0.02 (-0.09, 0.05) |
| *1 SD increment* |  | *0.02 (-0.03, 0.06)* | *-0.01 (-0.04, 0.01)* | *-0.00 (-0.03, 0.02)* |

^a^ Cognitive data have been converted to z-scores for the analysis.

Analysis adjusted for age, sex, education, ethnicity, waking time, time between waking and first measure of cortisol, seasonality, depressive symptoms, stress, Framingham cardiovascular risk score, coronary heart disease, stroke, diabetes, CVD medication, and antidepressants.

*p<0.05
